# Association Between Hospice Enrollment and Total Health Care Costs for Insurers and Families, 2002-2018

**DOI:** 10.1001/jamahealthforum.2021.5104

**Published:** 2022-02-11

**Authors:** Melissa D. Aldridge, Jaison Moreno, Karen McKendrick, Lihua Li, Ab Brody, Peter May

**Affiliations:** 1 Icahn School of Medicine at Mount Sinai, New York, New York; 2 James J. Peters Bronx VA Medical Center, Bronx, New York; 3 NYU Rory Meyers College of Nursing, New York, New York; 4 NYU Grossman School of Medicine, New York, New York; 5 Trinity College Dublin, Dublin, Ireland

## Abstract

**Importance:**

Use of hospice has been demonstrated to be cost saving to the Medicare program and yet the extent to which hospice saves money across all payers, including whether it shifts costs to families, is unknown.

**Objective:**

To estimate the association between hospice use and total health care costs including family out-of-pocket health care spending.

**Design, Setting, and Participants:**

This retrospective cohort study of health care spending in the last 6 months of life used data from the nationally representative Medicare Current Beneficiary Survey (MCBS) between the years 2002 and 2018. Participants were MCBS participants who resided in the community and died between 2002 and 2018.

**Exposures:**

Covariate balancing propensity scores were used to compare participants who used hospice (n = 2113) and those who did not (n = 3351), stratified by duration of hospice use.

**Main Outcomes and Measures:**

Total health care expenditures were measured across payers (family out-of-pocket, Medicare, Medicare Advantage, Medicaid, private insurance, private health maintenance organizations, Veteran’s Administration, and other) and by expenditure type (inpatient care, outpatient care, medical visits, skilled nursing, home health, hospice, durable medical equipment, and prescription drugs).

**Results:**

The study population included 5464 decedents (mean age 78.7 years; 48% female) and 38% enrolled with hospice. Total health care expenditures were lower for those who used hospice compared with propensity score weighted non-hospice control participants for the last 3 days of life ($2813 lower; 95% CI, $2396-$3230); last week of life ($6806 lower; 95% CI, $6261-$7350); last 2 weeks of life ($8785 lower; 95% CI, $7971-$9600); last month of life ($11 747 lower; 95% CI, $10 072-$13 422); and last 3 months of life ($10 908 lower; 95% CI, $7283-$14 533). Family out-of-pocket expenditures were lower for hospice enrollees in the last 3 days of life ($71; 95% CI, $43-$100); last week of life ($216; 95% CI, $175-$256); last 2 weeks of life ($265; 95% CI, $149-$382); and last month of life ($670; 95% CI, $530-$811) compared with those who did not use hospice. Health care savings were associated with reductions in inpatient care.

**Conclusions and Relevance:**

In this population-based cohort study of community-dwelling Medicare beneficiaries, hospice enrollment was associated with lower total health care costs for the last 3 days to 3 months of life. Importantly, we found no evidence of cost shifting from Medicare to families related to hospice enrollment. The magnitude of lower out-of-pocket spending to families who enrolled with hospice is meaningful to many Americans, particularly those with lower socioeconomic status.

## Key Points

**Question:**

Does hospice enrollment save money across all payers including families and does hospice shift costs from Medicare to families?

**Findings:**

In this cohort study, hospice use by community-dwelling Medicare beneficiaries was associated with significantly lower total health care costs across all payers in the last 3 days to last 3 months of life. We found no evidence of cost shifting from Medicare to families and families had significantly lower out-of-pocket health care costs in the last 3 days to last month of life when patients enrolled with hospice.

**Meaning:**

The findings of this study suggest that hospice care is associated with financial benefits to the health care system and families through lower health care costs at the end of life.

## Teaser

This cohort study estimates the association of hospice care with lower total health care costs for the last 3 days to 6 months of life.

## Introduction

Hospice has expanded to become the dominant model of home care for those with terminal illness and their families. Use of hospice has risen in the past 2 decades from 10% to 50%^1^ of Medicare decedents concurrent with the rise of in-home death and is considered to be an indicator of high-quality end-of-life care.^2,3^ Hospice is a comprehensive model of care that focuses on quality of life and provides an alternative to burdensome interventions.

Evidence from the early 2000s demonstrated that hospice was cost saving to the Medicare program.^4,5,6,7^ From 2002 to 2008, hospice use was found to save Medicare money across a range of hospice enrollment durations primarily owing to lower rates of hospital admission and in-hospital death for hospice users. Given that intensity of care at the end of life (outside of hospice) continues to rise,^2,3,8,9^ the cost savings to Medicare from hospice enrollment are likely even higher today.

A critical gap in this evidence, however, is how hospice use affects total health care costs, across all payers, including spending by patients and families. Use of hospice may shift economic burden onto families through higher out-of-pocket spending that may be required to care for patients at home. To the extent that hospice is not meeting patient needs adequately, families may face increased pressure to pay for supplemental care, services, medication, or other health care expenditures as has been found outside the hospice setting.^2,10,11,12,13^ The financial burden of family caregiving for hospice enrollees may be particularly high for patients with prolonged and substantial personal care needs (ie, those with advanced heart or lung disease or with dementia) and out-of-pocket expenditures for these populations can be substantial.^14,15,16,17,18^ Nevertheless, we know little about the drivers of costs to families of those at the end of life and whether hospice use provides health care savings in total, or merely shifts the financial burden from Medicare to families.

To address these questions, we used the Medicare Current Beneficiary Survey (MCBS), a nationally representative survey of Medicare beneficiaries, linked to Medicare administrative and claims data. We estimated total health care spending by payer (including family out-of-pocket, Medicare, Medicare Advantage, Medicaid, private insurance, private health maintenance organizations (HMOs), Veteran’s Administration, and other) at the end of life for hospice decedents compared with matched decedents who did not receive hospice. We estimated family health care spending using validated self-report of out-of-pocket spending. As hospice use increases and health care continues to shift from the hospital to community settings, the effect on family finances needs to be understood.

## Methods

### Study Population

We conducted a retrospective cohort study using data from the MCBS from 2002 through 2018. These data exclude survey results from 2014, which were not released by Centers for Medicare & Medicaid Services. The MCBS sample is representative of the Medicare population by age group with oversampling for the oldest old (85 and over), and includes Medicare Advantage enrollees. Of 9118 decedents, 8813 had spending data. We excluded individuals in nursing homes (n = 3059), as our focus is on community-based hospice use. We also excluded those who disenrolled from hospice prior to death (n = 290) because our outcomes are cumulative spending retrospectively from death and assignment to hospice vs no hospice for such individuals is not clear. Our final sample consisted of 5464 MCBS participants living in the community who died between 2002 and 2018 (eTable 1 in the Supplement). The Mount Sinai Institutional Review Board determined that this study was exempt secondary research for which patient informed consent was not required.

### Measures

Medicare Current Beneficiary Survey surveys are conducted in person, 3 times per year. All measures are self- or proxy-reported. The response rate for the MCBS in 2018 was 65.4%.^19^ All measured variables are as of an individual’s last MCBS interview date prior to death or the after-death proxy interview, which occurred an average of 69.6 days following death. We measured age at death, sex, education (college degree or less than college degree), marital status (married, not married), Medicaid coverage (yes/no), metropolitan area, and census region. Race was self-reported and categorized in MCBS as American Indian or Alaska Native, Asian, Black or African American, Native Hawaiian or Pacific Islander, and White. Ethnicity was self-reported and categorized as Hispanic (yes/no). We categorized race and ethnicity as Hispanic, Non-Hispanic Black, Non-Hispanic White, and Other/multiracial. We identified medical conditions using self-report of having ever had the illness and claims data diagnostic codes for heart disease, stroke, lung disease, cancer, and diabetes. For dementia, we used an inclusive case definition developed for use with MCBS data.^20^ We measured functional status based on self- or proxy-reported difficulty with 3 or more basic activities of daily living (ADLs): walking, feeding, dressing, toileting, bathing, and transferring.

We categorized hospice decedents by mutually exclusive periods of hospice enrollment based on the number of days prior to death that enrollment occurred, as follows: 0 to 7 days, 8 to 14 days, 15 to 28 days, 29 to 91 days, 92 to 182 days, and more than 182 days.

We measured total health care spending as the sum of family out-of-pocket, Medicare, Medicare Advantage, Medicaid, private insurance, private HMOs, Veteran’s Administration, and other. The MCBS team employs numerous strategies to improve the accuracy of self-reported spending data. Respondents are requested to record medical events on calendars provided by the interviewer and to save Explanation of Benefit forms from Medicare and receipts and statements from Medicaid and other public or private health insurers. To assist in reporting data on prescription medications, respondents are asked to bring to the interview bottles, tubes, and prescription bags provided by the pharmacy. All health care services paid for by Medicare are verified through linkage with Medicare claims.^21^ Family health care spending includes insurance deductibles, copays, prescription drugs, over-the-counter medications, medical devices and equipment, private duty nurses, social workers, and therapists. Expenditures were measured for the last 3 days, 1 week, 2 weeks, 1 month, 3 months, and 6 months of life. All costs were adjusted for inflation using the medical care component of the Consumer Price Index to 2018 dollars.

### Statistical Analysis

For each hospice enrollment period, we estimated covariate balancing propensity scores (CBPS)^22^ to estimate each decedent’s likelihood of hospice enrollment during the specified period (last 7, 8-14, 15-28, 29-91, 92-182, and >182 days of life). Variables in the CBPS were age, dementia, cancer, help with 3 or more ADLs, and region. Standardized differences are shown in eTable 2 in the Supplement. We used the CBPS-type weight in conjunction with the MCBS survey weights in all analytic comparisons.^22^ We used generalized linear models (GLMs) with a gamma distribution and log link function to analyze health care expenditures, adjusting for age, sex, race and ethnicity, education, marital status, survey year, Medicaid status, census region, metropolitan area, serious illness, and help with 3 or more ADLs. Decedents missing 1 or more covariates (n = 644) and were excluded from the analytic sample. We report the adjusted mean health care spending between groups of hospice enrollees and non-hospice control participants. We conducted sensitivity analyses stratifying by year of death (2002-2009 and 2010-2018) and including individuals who disenrolled from hospice in the hospice group.

## Results

The study population included 5464 community-dwelling decedents with mean age of 78.7 years at death representing 20 961 442 million Medicare beneficiary decedents. A total of 48% were female, 77.8% were non-Hispanic White, and 53.6% received help with 3 or more ADLs (Table 1). A total of 2113 (37.9%) decedents enrolled with hospice (median 12 days, mean 36 [SD 119] days). Hospice use by year is in eTable 3 in the Supplement.

**Table 1.** Characteristics of Medicare Decedents, 2002-2018^a^

Characteristic	%			*P* value
	Total (N = 5464)	Decedents who used hospice (n = 2113)	Decedents who did not use hospice (n = 3351)	
Age, mean (SD), y	78.7 (10.9)	81.2 (10.5)	77.1 (11.2)	<.001
Race/ethnicity				.001
Hispanic	6.5	6.3	6.5	
Non-Hispanic				
Black	10.2	7.6	11.8	
White	77.8	81.3	75.7	
Other/multiracial^b^	5.5	4.8	6.0	
Female sex	48.4	51.0	46.8	.01
Married	44.3	43.8	44.6	.59
Education: college degree	13.9	13.8	14.0	.89
Medicaid coverage	23.2	18.7	26.0	<.001
Serious illness				
Cancer	43.0	53.2	36.8	<.001
Dementia	30.4	38.4	25.5	<.001
Diabetes	35.9	32.9	37.7	.002
Heart disease	42.3	41.8	42.5	.66
Lung disease	33.1	32.2	33.7	.29
Stroke	22.7	22.4	22.8	.80
Receive help with ≥3 ADLs	53.6	62.6	48.2	<.001
Metropolitan area	76.0	80.1	73.5	.002
Region				.01
Northeast	18.4	14.6	20.7	
Midwest	23.2	25.0	22.1	
South	39.3	42.2	37.6	
West	19.1	18.2	19.6	

Abbreviation: ADLs, activities of daily living.

^a^ Table depicts characteristics of the study sample prior to propensity score weighting. All percentages incorporate Medicare Current Beneficiary Survey weights and weighted values exceed 1 million.

^b^ Other/Multiracial includes American Indian or Alaska Native, Asian, Native Hawaiian or Pacific Islander, and anyone who self-reported more than 1 race.

### Total Health Care Cost Savings Associated With Hospice Use

**Figure 1.**
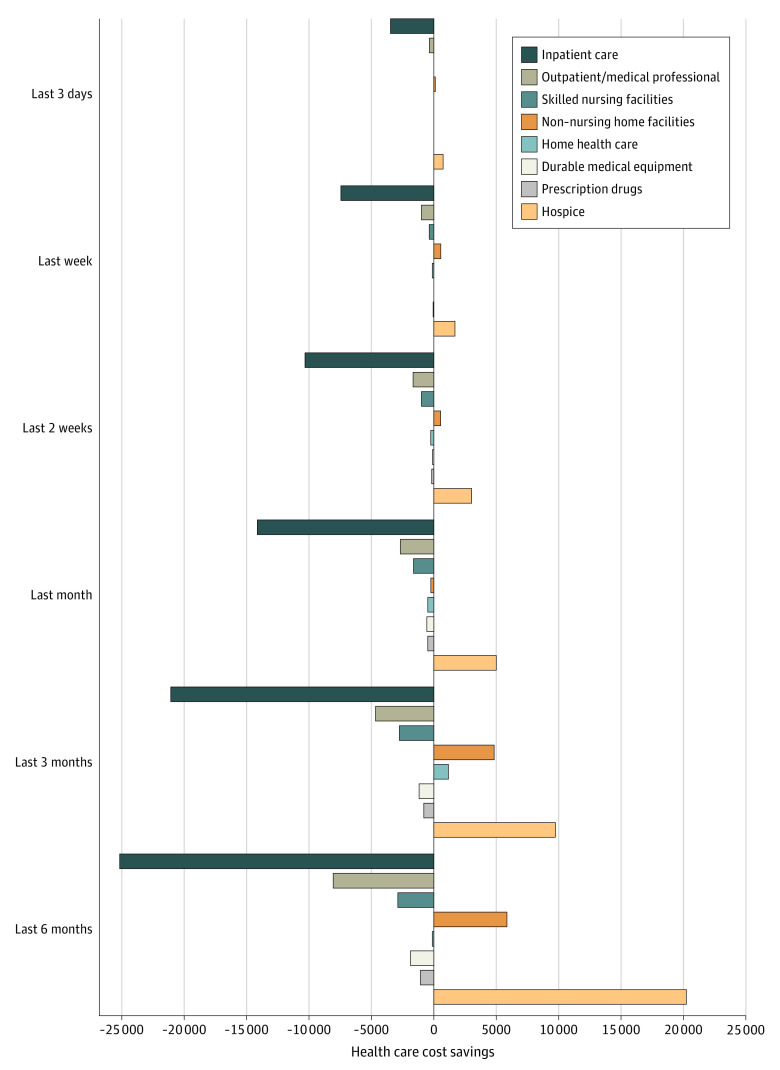
Adjusted Health Care Cost Savings for Individuals Enrolled With Hospice Compared With Non-Hospice Control Participants by Health Care Event, 2002-2018

Mean total (SD) health care costs in the last 3 days of life, week of life, 2 weeks of life, month of life, 3 months of life, and 6 months of life were $3879 ($6722), $7073 ($10 682), $10 874 ($15 331), $17 929 ($24 132), $29 048 ($36 916), and $40 843 ($46 747), respectively. Individuals who used hospice incurred significantly lower health care costs for the last 3 days of life ($2813 lower; 95% CI, $2396-$3230); last week of life ($6806 lower; 95% CI, $6261-$7350); last 2 weeks of life ($8785 lower; 95% CI, $7971-$9600); last month of life ($11 747 lower; 95% CI, $10 072-$13 422); and last 3 months of life ($10 908 lower; 95% CI, $7283-$14 533) compared with those who did not use hospice (Table 2). There was no significant difference in health care costs in the last 6 months of life between those who used hospice and those who did not. Health care cost savings for those who used hospice were driven by statistically significant reductions in expenditures for inpatient care ($3476 lower in the last 3 days of life; $7404 lower in the last week of life; $10 365 lower in the last 2 weeks of life; $14 175 lower in the last month of life; $21 047 lower in the last 3 months of life; and $24 953 lower in the last 6 months of life) (Figure 1). For each comparison group, differences in inpatient care were most apparent in the last week of life. Sensitivity analyses including individuals who disenrolled from hospice prior to death in the hospice group (eTable 4 in the Supplement) and stratifying by year of death (2002-2009 and 2010-2018) yielded similar results (eTable 5 in the Supplement).

**Table 2.** Adjusted Health Care Expenditures at the End of Life for Individuals Enrolled With Hospice and Non-Hospice Control Individuals, 2002-2018

Characteristic	Adjusted mean, $		Difference	*P* value
	Hospice group	Propensity score weighted controls		
**Total expenditures**				
Last 3 d^a^	2473	5285	−2831	<.001
Last wk^b^	2106	8911	−6806	<.001
Last 2 wks^c^	4083	12 869	−8785	<.001
Last mo^d^	8558	20 305	−11 747	<.001
Last 3 mos^e^	20 908	31 816	−10 908	<.001
Last 6 mos^f^	43 679	43 357	322	.93
**Family out of pocket**				
Last 3 d^a^	67	139	−71	<.001
Last wk^b^	46	262	−216	<.001
Last 2 wks^c^	159	424	−265	<.001
Last mo^d^	241	912	−670	<.001
Last 3 mos^e^	2412	1763	649	.41
Last 6 mos^f^	4096	2988	1109	.55
**Medicare**				
Last 3 d^a^	2121	4389	−2267	<.001
Last wk^b^	2029	7337	−5308	<.001
Last 2 wks^c^	3824	10 576	−6752	<.001
Last mo^d^	7835	16 559	−8724	<.001
Last 3 mos^e^	17 523	25 250	−7727	<.001
Last 6 mos^f^	36 208	33 036	3171	.26
**Private insurance**				
Last 3 d^a^	90	207	−117	<.001
Last wk^b^	3	347	−345	<.001
Last 2 wks^c^	11	567	−556	<.001
Last mo^d^	52	918	−866	<.001
Last 3 mos^e^	165	1499	−1334	<.001
Last 6 mos^f^	105	2252	−2147	<.001
**All other payers**				
Last 3 d^a^	231	568	−337	<.001
Last wk^b^	80	992	−912	<.001
Last 2 wks^c^	64	1408	−1344	<.001
Last mo^d^	213	2175	−1962	<.001
Last 3 mos^e^	500	3518	−3018	<.001
Last 6 mos^f^	1152	5422	−4270	<.001

Abbreviation: GLM, generalized linear models.

^a^ Sample sizes vary due to hospice enrollment period: hospice enrollment in the last week of life and comparison group (n = 3781).

^b^ Sample sizes vary due to hospice enrollment period: hospice enrollment 8-14 days before death and comparison group (n = 3242).

^c^ Sample sizes vary due to hospice enrollment period: hospice enrollment 15-28 days before death and comparison group (n = 3223).

^d^ Sample sizes vary due to hospice enrollment period: hospice enrollment 29-91 days before death and comparison group (n = 3202).

^e^ Sample sizes vary due to hospice enrollment period: hospice enrollment 92-182 days before death and comparison group (n = 2832).

^f^ Sample sizes vary due to hospice enrollment period: hospice enrollment >182 days before death and comparison group (n = 2551).

Variables included in the covariate balancing propensity score: age, dementia, cancer, help with 3+ activities of daily living, region. Variables included in the GLM model: age, sex, race/ethnicity, education, marital status, survey year, Medicaid status, census region, census metropolitan area, serious illness (dementia, heart disease, stroke, lung disease, cancer, and diabetes), and if the respondent needed help with 3 or more activities of daily living. All other payers includes Medicare Advantage, Medicaid, private health maintenance organizations, Veteran’s Administration, and other.

### Family Out-of-Pocket Health Care Cost Savings Associated With Hospice Use

**Figure 2.**
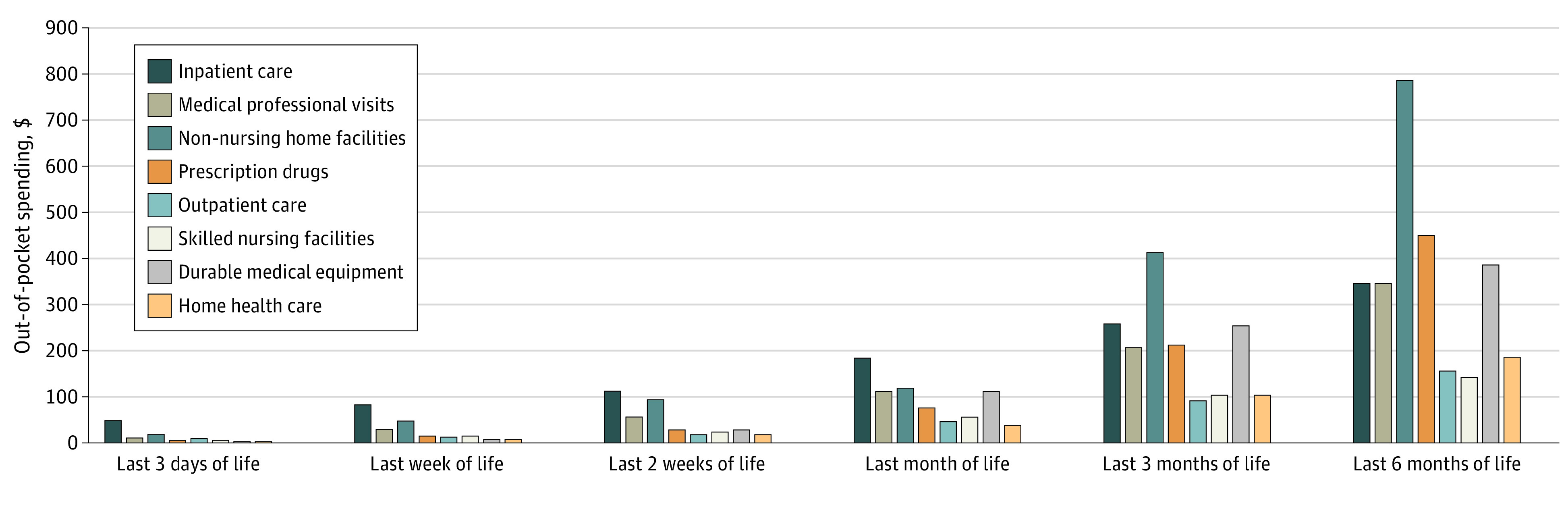
Family Out-of-Pocket Health Care Expenditures of the Entire Study Population at the End of Life (N = 5464), 2002-2018

Family out-of-pocket mean (SD) health care costs in the last 3 days of life, week of life, 2 weeks of life, month of life, 3 months of life, and 6 months of life were $106 ($521), $222 ($946), $388 ($1233), $883 ($2273), $1893 ($4185), and $3276 ($7097), respectively. Out-of-pocket spending in the last 3 days of life, last week of life, last 2 weeks of life, and last month of life were highest for inpatient care and in the last 3 and 6 months of life were highest for care in non-nursing home facilities (eg, in assisted living facilities) followed by costs for prescription drugs, durable medical equipment, and inpatient care (Figure 2).

Decedents who used hospice incurred significantly lower out-of-pocket costs for the last 3 days ($71 lower; 95% CI, $43-$100), 1 week ($216 lower; 95% CI, $175-$256), 2 weeks ($265 lower; 95% CI, $149-$382), and 1 month ($670 lower; 95% CI, $530-$811) of life (Table 2). There was no significant difference in out-of-pocket health care costs in the last 3 or 6 months of life between those who used hospice and those who did not.

### Medicare Cost Savings Associated With Hospice Use

Medicare costs in the last 3 days of life, week of life, 2 weeks of life, month of life, 3 months of life, and 6 months of life were $3187 (SD, $5902), $5785 (SD, $9169), $8910 (SD, $13 613), $14 520 (SD, $22 085), $22 798 (SD, $34 054), and $30 872 (SD, $42 742), respectively. Individuals who used hospice incurred significantly lower Medicare costs for the last 3 days ($2267 lower; 95% CI, $1864-$2671), last week ($5308 lower; 95% CI, $4771-$5845), last 2 weeks ($6752 lower; 95% CI, $5989-$7515), 1 month ($8724 lower; 95% CI, $7135-$10 313), and 3 months ($7727 lower; 95% CI, $4721-$10 733) of life (Table 2). There was no significant difference in Medicare costs associated with hospice enrollment for the last 6 months of life.

### Costs Savings Associated With Hospice Use for Private Insurance and All Other Payers

Private insurance expenditures were lower for those who enrolled with hospice compared with those who did not enroll with hospice for all periods examined (Table 2). Unlike Medicare and families, private insurance and all other payers combined (Medicare Advantage, Medicaid, private HMOs, Veteran’s Administration, and other) had evidence of cost savings associated with hospice in the last 6 months of life ($2147 lower for private insurance; 95% CI, $1905-$2388; and $4270 lower for all other payers; 95% CI, $3296-$5245).

## Discussion

To our knowledge, this is the first examination of the association between hospice use and total health care costs across all payers. We found that hospice use was associated with lower total health care costs in the last 3 days to last 3 months of life. Given that more than 80% of community-dwelling hospice enrollees in our sample received care for 3 months or less, cost savings are attributable to the vast majority of the community-dwelling hospice population. We found no difference in total health care expenditures in the last 6 months of life associated with hospice use.

Importantly, we found that use of hospice did not shift costs from Medicare to families through higher family out-of-pocket spending. Health care costs were lower for patients and families receiving hospice for each time period examined up to 1 month prior to death compared with health care costs of patients and families who did not receive hospice. The magnitude of out-of-pocket savings owing to hospice are meaningful to many Americans, particularly those with lower socioeconomic status, including the 23% in the present study sample who were Medicaid eligible. The estimated 1-month out-of-pocket savings associated with hospice is $670, which represents roughly 20% of the monthly income of the lowest third of older adults in the US.^23^ Further, the $670 estimated savings represents an almost 75% reduction in out-of-pocket costs compared with older adults who did not receive hospice care.

The present study provides new details regarding family out-of-pocket spending at the end of life. In the last month of life, families paid the highest amount in out-of-pocket health care expenses for inpatient care compared with what they spent for outpatient care, medical provider visits, prescription drugs, and other health care needs. In the last 3 months and 6 months of life, family health care spending was driven by care received in non-nursing home facilities such as assisted living facilities. Family spending for health care in these facilities averages $413 in the last 3 months of life and $789 in the last 6 months of life. These types of community-based residential settings comprise a wide range of environments with differing amounts of built-in services and high rates of hospice use.^24^ The type of health care received in these settings and the financial burden for those residing there is an important emerging area of research.

Hospice was associated with lower total health care expenditures across all payers and families primarily owing to lower spending for inpatient care, consistent with prior work.^4^ A primary goal of hospice is to manage pain and other symptoms in the home setting and avoid hospitalization. Exacerbations in clinical conditions can be addressed through higher levels of hospice care including continuous home care, which provides a minimum of 8 hours of licensed nursing care per day in the home. Although use of continuous home care by hospices is more expensive to Medicare, its association with reductions in hospitalizations may be contributing to our finding of health care savings.^25^

Medicare incurred lower health care costs for all measured time periods up to 3 months prior to death for community-dwelling individuals who enrolled with hospice. Cost savings were evident even for those who only enrolled with hospice in their last week of life, which is the case for approximately 25% of all hospice users in the US.^1^ Although even a single day of hospice care may be beneficial to patients and families, many advocate for patients to receive at least 2 weeks of hospice care to experience the benefits. Greater cost savings from longer enrollment align with this quality metric. For those who enroll with hospice for 6 months of more prior to death, the cost of hospice care itself offsets the reductions in inpatient spending, mostly owing to high inpatient costs that occur near the end of life.

It will be important to evaluate the effect of the 2016 hospice payment reform on Medicare hospice spending. The 2-tiered per diem payment methodology implemented in 2016 pays higher per diem amounts for the first 60 days of hospice care and lower per diem amounts for each day beyond 60 days, as well as a service intensity add-on payment for visits in the last week of life. Although this change does not effect family out-of-pocket spending or spending by insurers other than Medicare, its effect on Medicare spending, differentially across length of hospice enrollment category, is a key area for future research.

### Limitations

The present study limitations include the inability to adjust for unmeasured characteristics of those who do and do not use hospice. In particular, preferences among people with serious illness, their family members, and their health care professionals are likely associated with both the exposure and outcome of interest. Given that preferences are not measured in MCBS or any of our linked data, we were unable to control for them. Although tools such as instrumental variables could help address unmeasured confounders such as preferences for care,^26^ we did not identify a valid instrument in the data set. While imperfect, propensity score weighting is among the most rigorous tools available to compare groups outside of a randomized trial and it has been used in a wide range of studies to inform policy-relevant questions with observational data. Our analyses yield important, new information regarding spending at end of life across all payers, including families, in a large, population-based sample that could not otherwise be achieved for ethical and practical reasons with a randomized trial design. Second, we include only monetary costs and do not include unpaid caregiving by family members, which may be higher for those who are not receiving the interdisciplinary care of hospice teams. In addition, owing to sample size limitations, our examination of expenditures in the last 3 days of life is for hospice users who enrolled with hospice 0 to 7 days prior to death and therefore inflates the expenditures associated with hospice for those who enrolled with hospice 0 to 2 days prior to death. Despite this conservative approach, hospice use was associated with cost savings for all payers in the last 3 days of life. Finally, we are unable to account for payments that Medicare receives from hospices owing to the Hospice Aggregate Cap, which 10% to 15% of hospices incur each year.^27,28^ Its inclusion would decrease estimates of Medicare spending for hospice enrollees and increase cost savings owing to hospice enrollment.

## Conclusions

The findings of this cohort study suggest that hospice use is an example of a health care model that demonstrates both components of the value proposition: it improves the quality of end-of-life care and is associated with lower health care costs. Moreover, unlike many other aspects of our health care system, cost reductions to insurers in the present study did not translate into higher costs for patients and their families.

## Notes

Supplement. **eTable 1. **Sample Derivation, Medicare Current Beneficiary Survey, 2002-2018 **eTable 2. **Standardized Differences between the Hospice and No Hospice Groups Before and After Propensity Score Weighting **eTable 3. **Hospice Use for Sampled Community-Dwelling Medicare Current Beneficiary Survey Participants, 2002-2018 **eTable 4. **Adjusted Healthcare Expenditures at the End of Life for Individuals Enrolled with Hospice and Non-Hospice Controls, 2002-2018, *including those who disenrolled from hospice in the hospice group* **eTable 5. **Adjusted Healthcare Expenditures at the End of Life for Individuals Enrolled with Hospice and Non-Hospice Controls, 2002-2009 and 2010-2018
