# Supplementary material for: Association Between Hospice Enrollment and Total Health Care Costs for Insurers and Families, 2002-2018
Source: JAMA Health Forum. 2022 Feb 11;3(2):e215104. doi: 10.1001/jamahealthforum.2021.5104 (PMC8903119; doi:10.1001/jamahealthforum.2021.5104)
Supplement: Supplement. — eTable 1. Sample Derivation, Medicare Current Beneficiary Survey, 2002-2018 eTable 2. Standardized Differences between the Hospice and No Hospice Groups Before and After Propensity Score Weighting eTable 3. Hospice Use for Sampled Community-Dwelling Medicare Current Beneficiary Survey Participants, 2002-2018 eTable 4. Adjusted Healthcare Expenditures at the End of Life for Individuals Enrolled with Hospice and Non-Hospice Controls, 2002-2018, including those who disenrolled from hospice in the hospice group eTable 5. Adjusted Healthcare Expenditures at the End of Life for Individuals Enrolled with Hospice and Non-Hospice Controls, 2002-2009 and 2010-2018 [file jamahealthforum-e215104-s001.pdf]

Supplemental Online Content

Aldridge MD, Moreno J, McKendrick K, Li L, Brody A, May P. Association between hospice enrollment and total health care costs for insurers and families, 2002-2018. *JAMA Health Forum*. 2022;3(2):e215104. doi:10.1001/jamahealthforum.2021.5104

eTable 1. Sample Derivation, Medicare Current Beneficiary Survey, 2002-2018

eTable 2. Standardized Differences between the Hospice and No Hospice Groups Before and After Propensity Score Weighting

eTable 3. Hospice Use for Sampled Community-Dwelling Medicare Current Beneficiary Survey Participants, 2002-2018

eTable 4. Adjusted Healthcare Expenditures at the End of Life for Individuals Enrolled with Hospice and Non-Hospice Controls, 2002-2018, *including those who disenrolled from hospice in the hospice group*

eTable 5. Adjusted Healthcare Expenditures at the End of Life for Individuals Enrolled with Hospice and Non-Hospice Controls, 2002-2009 and 2010-2018

This supplemental material has been provided by the authors to give readers additional information about their work.

© 2022 Aldridge et al. *JAMA Health Forum.*

eTable 1. Sample Derivation, Medicare Current Beneficiary Survey, 2002-2018

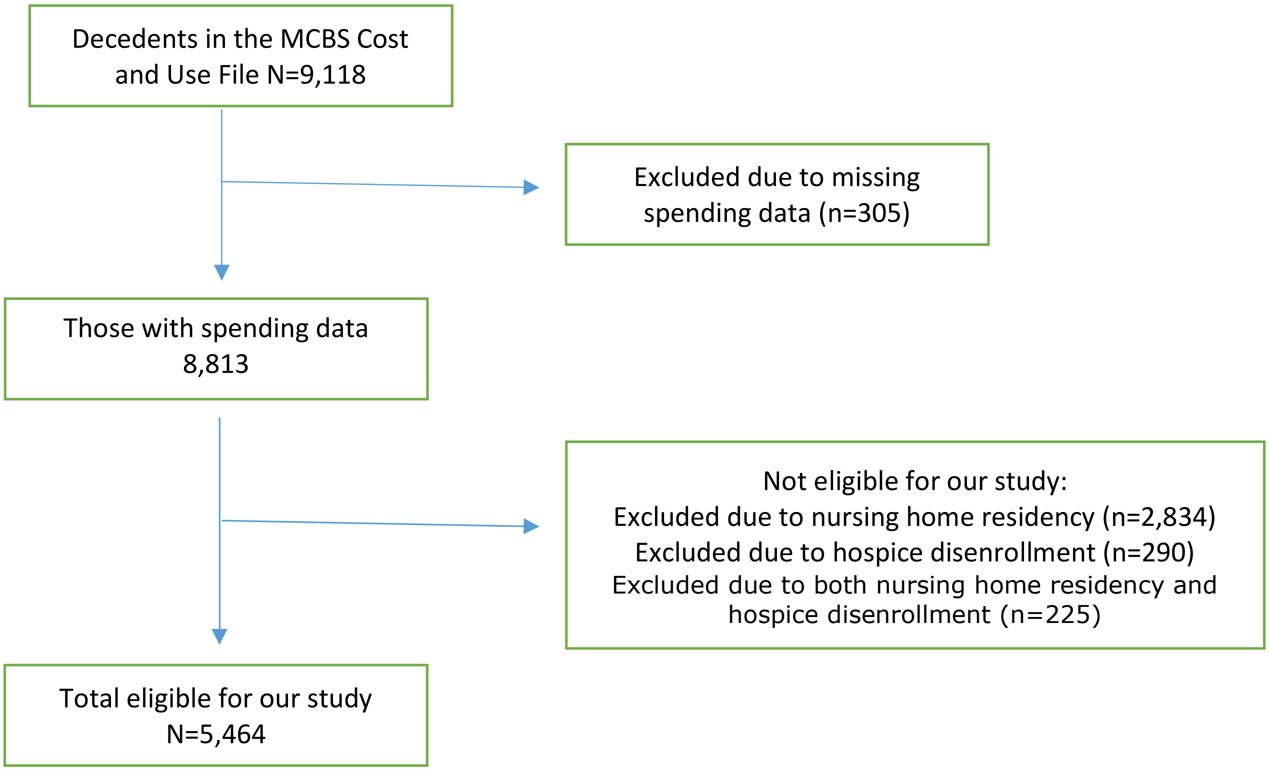

© 2022 Aldridge et al. *JAMA Health Forum.*

eTable 2. Standardized Differences between the Hospice and No Hospice Groups Before and After Propensity Score Weighting

	Before Propensity Score Weighting			After Propensity Score Weighting		
Group	Hospice	No Hospice	Std	Hospice	No Hospice	Std
Variable	Mean	Mean	diff	Mean	Mean	diff
**Last week**						
Dementia[p]	0.37	0.29	0.167	0.31	0.31	-0.009
Region: northeast[p]	0.13	0.20	-0.206	0.18	0.19	-0.006
Region: midwest[p]	0.26	0.23	0.065	0.24	0.23	0.003
Region: south[p]	0.42	0.38	0.065	0.39	0.39	0.000
Region: west[p]	0.20	0.18	0.042	0.19	0.19	0.003
Age[p]	82.26	78.94	0.304	79.94	79.61	0.030
ADL help 3+[p]	0.62	0.49	0.278	0.52	0.52	0.000
Cancer[p]	0.51	0.37	0.284	0.40	0.40	0.004
Female sex	0.49	0.49	0.003	0.50	0.50	-0.003
Non-Hispanic white	0.81	0.77	0.107	0.80	0.77	0.070
Less than high school	0.36	0.39	-0.068	0.37	0.39	-0.058
Married	0.44	0.41	0.058	0.44	0.41	0.057
Medicaid	0.21	0.27	-0.145	0.24	0.27	-0.071
Metro	0.78	0.71	0.173	0.78	0.70	0.181
Heart disease	0.43	0.42	0.013	0.42	0.43	-0.025
Stroke	0.23	0.23	-0.014	0.22	0.24	-0.030
Lung disease	0.32	0.31	0.011	0.31	0.32	-0.008
Diabetes	0.37	0.35	0.051	0.37	0.35	0.051
**Last two weeks**						
Dementia[p]	0.43	0.29	0.290	0.31	0.30	0.020
Region: northeast[p]	0.14	0.20	-0.158	0.20	0.20	-0.005
Region: midwest[p]	0.25	0.23	0.057	0.23	0.23	0.007
Region: south[p]	0.45	0.38	0.140	0.39	0.39	0.007
Region: west[p]	0.15	0.18	-0.092	0.18	0.18	-0.013
Age[p]	83.11	78.94	0.382	79.47	79.27	0.018
ADL help 3+[p]	0.63	0.49	0.288	0.50	0.50	0.003
Cancer[p]	0.51	0.37	0.281	0.38	0.38	0.002
Female sex	0.49	0.49	-0.008	0.46	0.50	-0.071
Non-Hispanic white	0.84	0.77	0.182	0.79	0.77	0.045
Less than high school	0.40	0.39	0.008	0.39	0.39	0.000
Married	0.41	0.41	0.002	0.43	0.41	0.028
Medicaid	0.15	0.27	-0.305	0.14	0.27	-0.309
Metro	0.80	0.71	0.214	0.84	0.71	0.305
Heart disease	0.38	0.42	-0.097	0.35	0.43	-0.161
Stroke	0.25	0.23	0.041	0.24	0.23	0.021
Lung disease	0.31	0.31	0.001	0.28	0.31	-0.070
Diabetes	0.33	0.35	-0.041	0.32	0.35	-0.044
**Last month**						

© 2022 Aldridge et al. *JAMA Health Forum.*

Dementia[p]	0.41	0.29	0.238	0.31	0.31	-0.002
Region: northeast[p]	0.12	0.20	-0.225	0.19	0.20	-0.017
Region: midwest[p]	0.23	0.23	-0.009	0.23	0.23	0.009
Region: south[p]	0.48	0.38	0.193	0.39	0.39	-0.001
Region: west[p]	0.17	0.18	-0.025	0.18	0.18	0.008
Age[p]	81.94	78.94	0.271	80.11	79.18	0.085
ADL help 3+[p]	0.61	0.49	0.259	0.50	0.50	0.007
Cancer[p]	0.52	0.37	0.298	0.38	0.38	-0.015
Female sex	0.50	0.49	0.018	0.50	0.49	0.002
Non-Hispanic white	0.80	0.77	0.073	0.81	0.77	0.111
Less than high school	0.36	0.39	-0.062	0.39	0.39	-0.015
Married	0.48	0.41	0.125	0.48	0.41	0.140
Medicaid	0.19	0.27	-0.193	0.22	0.27	-0.130
Metro	0.79	0.71	0.186	0.80	0.71	0.209
Heart disease	0.39	0.42	-0.077	0.38	0.43	-0.100
Stroke	0.23	0.23	-0.004	0.21	0.23	-0.062
Lung disease	0.26	0.31	-0.120	0.26	0.32	-0.132
Diabetes	0.24	0.35	-0.241	0.24	0.35	-0.243
**Last three months**						
Dementia[p]	0.42	0.29	0.267	0.31	0.31	-0.003
Region: northeast[p]	0.14	0.20	-0.175	0.20	0.20	0.015
Region: midwest[p]	0.28	0.23	0.117	0.23	0.23	-0.009
Region: south[p]	0.41	0.38	0.053	0.40	0.39	0.020
Region: west[p]	0.17	0.18	-0.028	0.17	0.18	-0.029
Age[p]	82.43	78.94	0.318	80.29	79.23	0.097
ADL help 3+[p]	0.60	0.49	0.227	0.50	0.50	-0.004
Cancer[p]	0.52	0.37	0.312	0.39	0.38	0.021
Female sex	0.55	0.49	0.118	0.56	0.49	0.138
Non-Hispanic white	0.85	0.77	0.209	0.82	0.77	0.140
Less than high school	0.34	0.39	-0.118	0.35	0.39	-0.090
Married	0.44	0.41	0.055	0.42	0.41	0.023
Medicaid	0.19	0.27	-0.198	0.20	0.27	-0.168
Metro	0.78	0.71	0.178	0.80	0.71	0.218
Heart disease	0.39	0.42	-0.059	0.39	0.43	-0.065
Stroke	0.20	0.23	-0.071	0.17	0.23	-0.154
Lung disease	0.30	0.31	-0.038	0.30	0.31	-0.039
Diabetes	0.25	0.35	-0.207	0.23	0.35	-0.247
**Last six months**						
Dementia[p]	0.51	0.29	0.454	0.30	0.30	-0.016
Region: northeast[p]	0.17	0.20	-0.092	0.20	0.20	0.002
Region: midwest[p]	0.24	0.23	0.037	0.23	0.23	0.003
Region: south[p]	0.40	0.38	0.024	0.39	0.39	0.009
Region: west[p]	0.19	0.18	0.021	0.18	0.18	-0.017
Age[p]	83.75	78.94	0.447	78.56	79.23	-0.062
ADL help 3+[p]	0.71	0.49	0.466	0.50	0.50	-0.010

© 2022 Aldridge et al. *JAMA Health Forum.*

Cancer[p]	0.45	0.37	0.167	0.38	0.38	0.005
Female sex	0.61	0.49	0.237	0.58	0.50	0.177
Non-Hispanic white	0.87	0.77	0.271	0.78	0.77	0.043
Less than high school	0.37	0.39	-0.040	0.37	0.39	-0.059
Married	0.44	0.41	0.058	0.49	0.41	0.161
Medicaid	0.16	0.27	-0.272	0.14	0.27	-0.315
Metro	0.77	0.71	0.145	0.77	0.71	0.139
Heart disease	0.40	0.42	-0.038	0.36	0.43	-0.141
Stroke	0.26	0.23	0.065	0.22	0.23	-0.038
Lung disease	0.31	0.31	-0.002	0.34	0.31	0.063
Diabetes	0.29	0.35	-0.122	0.30	0.35	-0.108
**More than six months**						
Dementia[p]	0.55	0.29	0.536	0.30	0.32	-0.031
Region: northeast[p]	0.12	0.20	-0.226	0.20	0.20	0.007
Region: midwest[p]	0.21	0.23	-0.033	0.23	0.23	0.009
Region: south[p]	0.46	0.38	0.158	0.38	0.39	-0.015
Region: west[p]	0.20	0.18	0.048	0.18	0.18	0.002
Age[p]	85.65	78.94	0.654	79.60	79.39	0.020
ADL help 3+[p]	0.72	0.49	0.501	0.51	0.51	0.013
Cancer[p]	0.50	0.37	0.274	0.39	0.38	0.013
Female sex	0.62	0.49	0.252	0.62	0.50	0.256
Non-Hispanic white	0.85	0.77	0.201	0.82	0.77	0.145
Less than high school	0.45	0.39	0.113	0.50	0.39	0.206
Married	0.30	0.41	-0.249	0.31	0.41	-0.203
Medicaid	0.23	0.27	-0.100	0.19	0.27	-0.185
Metro	0.80	0.71	0.213	0.82	0.71	0.273
Heart disease	0.46	0.42	0.067	0.42	0.43	-0.010
Stroke	0.26	0.23	0.070	0.14	0.23	-0.210
Lung disease	0.28	0.31	-0.070	0.27	0.31	-0.097
Diabetes	0.23	0.35	-0.248	0.25	0.35	-0.213

[p] Variables included in the propensity model.

© 2022 Aldridge et al. *JAMA Health Forum.*

eTable 3. Hospice Use for Sampled Community-Dwelling Medicare Current Beneficiary Survey Participants, 2002-2018

	Proportion of Decedents Enrolled with Hospice
2002-2005	28.3%
2006-2009	33.3%
2010-2013	41.5%
2015-2018	46.8%

Note: Medicare Current Beneficiary Survey data were not released by Centers for Medicare & Medicaid Services for 2014.

© 2022 Aldridge et al. *JAMA Health Forum.*

eTable 4. Adjusted Healthcare Expenditures at the End of Life for Individuals Enrolled with Hospice and Non-Hospice Controls, 2002-2018, *including those who disenrolled from hospice in the hospice group*

	Hospice Group Adjusted Mean $	Propensity Score Weighted Controls Adjusted Mean $	Difference	P-value
**Total Expenditures**				
Last 3 days[a]	2,465	5,286	-2,821	<.001
Last week[b]	2,379	8,911	-6,533	<.001
Last 2 weeks[c]	4,365	12,879	-8,514	<.001
Last month[d]	9,626	20,333	-10,707	<.001
Last 3 months[e]	22,549	31,886	-9,337	<.001
Last 6 months[f]	44,431	43,461	970	.82
**Family Out of Pocket**				
Last 3 days[a]	66	139	-73	<.001
Last week[b]	54	260	-206	<.001
Last 2 weeks[c]	141	425	-284	<.001
Last month[d]	258	910	-652	<.001
Last 3 months[e]	2,119	1,773	346	.53
Last 6 months[f]	4,068	2,974	1,094	.26
**Medicare**				
Last 3 days[a]	2,119	4388	-2,269	<.001
Last week[b]	2,286	7,341	-5,055	<.001
Last 2 weeks[c]	4,094	10,589	-6,495	<.001
Last month[d]	8,805	16,589	-7,784	<.001
Last 3 months[e]	19,070	25,294	-6,224	<.001
Last 6 months[f]	36,086	33,157	2,929	.39
**Private Insurance**				
Last 3 days[a]	88	207	-119	<.001
Last week[b]	5	355	-350	<.001
Last 2 weeks[c]	15	551	-536	<.001
Last month[d]	80	919	-839	<.001
Last 3 months[e]	236	1,495	-1,259	<.001
Last 6 months[f]	629	2,336	-1,707	<.001
**All Other Payers**				
Last 3 days[a]	228	569	-340	<.001
Last week[b]	89	982	-894	<.001
Last 2 weeks[c]	77	1,418	-1,341	<.001
Last month[d]	277	2,150	-1,873	<.001
Last 3 months[e]	775	3,589	-2,814	<.001
Last 6 months[f]	1,889	5,594	-3,705	<.001

Notes:

Variables included in the covariate balancing propensity score: age, dementia, cancer, help w 3+ ADLs, region; Variables included in the GLM model: age, sex, race/ethnicity, education, marital status, survey year, Medicaid status, census region, census metropolitan area, serious

© 2022 Aldridge et al. *JAMA Health Forum.*

illness (dementia, heart disease, stroke, lung disease, cancer, and diabetes), and if the respondent needed help with 3 or more ADLs.

Sample sizes vary due to hospice enrollment period: [a] *hospice enrollment in the last week of life and comparison group (N=3,803);* [b] *hospice enrollment 8-14 days before death and comparison group (N=3,258);* [c] *hospice enrollment 15-28 days before death and comparison group (N=3,240);* [d] *hospice enrollment 29-91 days before death and comparison group (N=3,230);* [e] *hospice enrollment 92-182 days before death and comparison group (N=2,865);* [f] *hospice enrollment >182 days before death and comparison group (N=2,666).*

All Other Payers includes Medicare Advantage, Medicaid, private HMOs, Veteran's Administration, and other.

© 2022 Aldridge et al. *JAMA Health Forum.*

eTable 5. Adjusted Healthcare Expenditures at the End of Life for Individuals Enrolled with Hospice and Non-Hospice Controls, 2002-2009 and 2010-2018

	2002-2009				2010-2018			
	Hospice Group Adjusted Mean $	Propensity Score Weighted Controls Adjusted Mean $	Difference		Hospice Group Adjusted Mean $	Propensity Score Weighted Controls Adjusted Mean $	Difference	
**Total**								
Last 3 days[a]	2,466	5,270	-2,804	**	2,507	5,307	-2,799	**
Last week[b]	1,830	8,983	-7,153	**	2,397	8,788	-6,391	**
Last 2 weeks[c]	3,770	13,339	-9,569	**	4,439	12,166	-7,727	**
Last month[d]	8,602	21,151	-12,549	**	8,559	18,995	-10,436	**
Last 3 months[e]	19,551	32,056	-12,505	**	21,908	31,452	-9,543	**
Last 6 months[f]	39,368	43,084	-3,717		56,437	43,832	12,605	
**Family Out of Pocket**								
Last 3 days[a]	79	138	-59	*	53	144	-92	**
Last week[b]	57	269	-212	**	35	253	-218	**
Last 2 weeks[c]	182	444	-262	*	116	410	-295	**
Last month[d]	244	936	-692	**	215	917	-702	**
Last 3 months[e]	949	1,840	-891	*	3,039	1,697	1,342	
Last 6 months[f]	1,673	3,074	-1,401	*	7,727	2,936	4,791	
**Medicare**								
Last 3 days[a]	2,112	4,379	-2,267	**	2,190	4,404	-2,214	**
Last week[b]	1,722	7,420	-5,697	**	2,440	7,221	-4,782	**
Last 2 weeks[c]	3,488	11,004	-7,516	**	4,411	9,953	-5,542	**
Last month[d]	8,080	17,287	-9,207	**	7,880	15,438	-7,558	**
Last 3 months[e]	17,239	25,534	-8,295	**	17,535	24,919	-7,384	**
Last 6 months[f]	35,486	32,736	2,749		42,747	33,666	9,081	
**Private**								
Last 3 days[a]	101	195	-94	*	77	291	-214	**
Last week[b]	1	385	-383	**	4	417	-413	**
Last 2 weeks[c]	23	573	-550	**	3	885	-882	**
Last month[d]	36	1,066	-1,029	**	77	978	-901	**
Last 3 months[e]	85	1,782	-1,697	**	207	1,587	-1,380	**
Last 6 months[f]	158	2,711	-2,554	**	49	2,033	-1,984	**
**All Other Payers**								
Last 3 days[a]	185	583	-398	**	243	582	-339	*
Last week[b]	51	991	-940	**	90	1,033	-943	**
Last 2 weeks[c]	24	1,346	-1,322	**	110	1,438	-1,328	**
Last month[d]	104	2,017	-1,913	**	252	2,382	-2,130	**
Last 3 months[e]	552	3,204	-2,652	**	435	3,989	-3,554	**
Last 6 months[f]	1,291	5,040	-3,749	**	1,057	5,873	-4,816	**

Notes:
Variables included in the covariate balancing propensity score: age, dementia, cancer, help w 3+ ADLs, region; Variables included in the GLM model: age, sex, race/ethnicity, education, marital status, survey year, Medicaid status, census region, census metropolitan area, serious illness (dementia, heart disease, stroke, lung disease, cancer, and diabetes), and if the respondent needed help with 3 or more ADLs.

2002-2009: Sample sizes vary due to hospice enrollment period: [a] hospice enrollment in the last week of life and comparison group (N=2,422); [b] hospice enrollment 8-14 days before death and comparison group (N=2,140); [c] hospice enrollment 15-28 days before death and comparison group (N=2,139); [d] hospice enrollment 29-91 days before death and comparison group (N=2,120); [e] hospice enrollment 92-182 days before death and comparison group (N=1,885); [f] hospice enrollment >182 days before death and comparison group (N=1,710).

2010-2018: Sample sizes vary due to hospice enrollment period: [a] hospice enrollment in the last week of life and comparison group (N=1,359); [b] hospice enrollment 8-14 days before death and comparison group (N=1,102); [c] hospice enrollment 15-28 days before death and comparison group (N=1,084); [d] hospice enrollment 29-91 days before death and comparison group (N=1,082); [e] hospice enrollment 92-182 days before death and comparison group (N=947); [f] hospice enrollment >182 days before death and comparison group (N=841).

All Other Payers includes Medicare Advantage, Medicaid, private HMOs, Veteran's Administration, and other.

** p<0.05 ** p<0.001*

© 2022 Aldridge et al. *JAMA Health Forum.*
